# Tunable Nanoantennas for Surface Enhanced Infrared Absorption Spectroscopy by Colloidal Lithography and Post-Fabrication Etching

**DOI:** 10.1038/srep44069

**Published:** 2017-03-08

**Authors:** Kai Chen, Thang Duy Dao, Tadaaki Nagao

**Affiliations:** 1 International Center for Materials Nanoarchitectonics (MANA), National Institute for Materials Science (NIMS), 1-1 Namiki, Tsukuba, Ibaraki 305-0044, Japan; 2 CREST, Japan Science and Technology Agency, 4-1-8 Honcho, Kawaguchi, Saitama, 332-0012, Japan; 3 Department of Condensed Matter Physics, Graduate School of Science, Hokkaido University, Sapporo 060-0810, Japan

## Abstract

We fabricated large-area metallic (Al and Au) nanoantenna arrays on Si substrates using cost-effective colloidal lithography with different micrometer-sized polystyrene spheres. Variation of the sphere size leads to tunable plasmon resonances in the middle infrared (MIR) range. The enhanced near-fields allow us to detect the surface phonon polaritons in the natural SiO_2_ thin layers. We demonstrated further tuning capability of the resonances by employing dry etching of the Si substrates with the nanoantennas acting as the etching masks. The effective refractive index of the nanoantenna surroundings is efficiently decreased giving rise to blueshifts of the resonances. In addition, partial removal of the Si substrates elevates the nanoantennas from the high-refractive-index substrates making more enhanced near-fields accessible for molecular sensing applications as demonstrated here with surface-enhanced infrared absorption (SEIRA) spectroscopy for a thin polymer film. We also directly compared the plasmonic enhancement from the Al and Au nanoantenna arrays.

Surface-enhanced spectroscopy utilizes enhanced near-field near rough surface or rationally designed nanoantennas to boost the scattering or absorption signals from the attached molecules significantly improving the detection limit even down to single-molecule level^1^,^2^,^3^,^4^. Two versions of this technique, surface-enhanced Raman spectroscopy (SERS) and surface-enhanced infrared absorption (SEIRA) spectroscopy, have been extensively studied, especially SERS, for a variety of sensing applications^3^,^4^,^5^,^6^,^7^. Since most molecules have their characteristic absorption bands in the infrared wavelength range, infrared absorption spectroscopy has been one of the most powerful methods for the identification of unknown organic species. Therefore, SEIRA provides a powerful and reliable tool to detect tiny amount of hazardous materials as well as to study conformation change and molecular interactions of biomolecules^1^,^2^,^8^,^9^,^10^,^11^,^12^,^13^.

Together with the effect of significantly enhanced near-field, optical nanoantennas have the advantage that the resonances of rationally designed nanoantennas can be precisely tuned to certain wavelength range to match with specific vibrational modes that are to be detected^14^,^15^,^16^,^17^,^18^. Most of these nanoantennas are fabricated by e-beam lithography (EBL) or focused-ion beam (FIB) that have the capability of creating complex nanostructures with good fidelity. However, the operation of EBL or FIB tends to be expensive and time-consuming. Therefore, alternative techniques have been proposed such as direct laser writing (DLW)^11^, nanospherical-lens lithography (NLL)^19^, and colloidal lithography (CL)^20^. CL, also known as nanosphere lithography (NSL)^21^,^22^, is a scalable and cost-effective technique that can generate a variety of metallic nanoparticles^23^,^24^, even sophisticated metasurfaces when combined with other lithographic techniques such as dry etching^25^,^26^ or angled-deposition^27^,^28^. The simplest version of CL uses close-packed colloidal sphere monolayers as deposition masks to create triangular nanoparticle arrays on the substrates underneath. Varying the sizes of the spheres can systematically tune the dimension of the nanotriangles and their corresponding plasmon resonances. This basic version of CL has been widely used with nanoscale spheres in the visible wavelength range for a variety of applications^29^,^30^,^31^,^32^. However, there are only a few reports of using triangular nanoparticles for infrared applications. Micrometer-sized spheres are needed to bring the nanotriangle resonance to the infrared range^8^,^9^,^33^. In particular, Hoffmann *et al*. have demonstrated widely tunable plasmon resonances from Au nanotriangles by using different substrate materials and showed their suitability for applications in SEIRA with measurement area of a few hundred μm^2^ that is nearly defect-free within the region^8^. While this method enables wide tuning range, it is highly desirable that the resonances can be continuously and precisely tuned to ensure excellent spectral overlap with certain molecular vibration frequencies. To this end, it is noted that the plasmon resonances of the nanoparticles can also be readily tuned by post-fabrication partial etching of the substrates to reduce the influence of the polarizability of the substrates^34^,^35^.

Here, we use CL to fabricate large-area metallic (Al and Au) infrared nanoantennas over several mm^2^ on Si substrates and subsequently tune the plasmon resonances by etching the Si substrates using CF_4_ plasma with the nanoantennas as etching masks. The etching reduces the effective refractive index of the environment of the nanoantennas and enables blue-shift of the plasmon resonances. Furthermore, the etching increases the accessible volume of enhanced near-field, part of which is freed from the Si substrate, as well as the packing volume for the molecules in sensing applications. Due to the presence of the natural oxide SiO_2_, we observed plasmon-enhanced surface phonon polaritons from the SiO_2_ layer. In addition, we directly compared the SEIRA performances of Al and Au nanoantennas that were fabricated by CL. Gold nanoantennas provide better signals, nearly 2 times larger than those from Al nanoantennas that are covered with the natural oxide Al_2_O_3_ layer. This signal enhancement difference should be taken into account, together with the cost of the materials, for nanoantenna design for surface-enhanced spectroscopies.

## Results and Discussions

We first demonstrate the tunability of the nanoantennas by using microspheres with different sizes. In the basic version of CL, monolayers of polymer spheres are used as deposition masks. In the perfect case with a single-crystal monolayer, the size of the nanotriangle can be approximated using the following simple formula^21^:


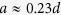


where *a* is the perpendicular bisector of the nanotriangle and *d* is the diameter of the microsphere. The plasmon resonances of the metallic nanotriangles can thus be readily tuned across a wide wavelength range simply by changing the sizes of the spheres, which are commercially available with diameters ranging from tens of nanometers to several micrometers. Although this tunability has been demonstrated in many reports in the visible wavelength range, its discussions and applications in the IR range are still very limited^8^,^9^,^33^. Here we employed various microspheres to tune the corresponding plasmon modes to the spectral region-of-interest for molecular sensing. The monolayers of polystyrene microspheres were fabricated on Si chips using the same method detailed in our previous publication^25^.

**Figure 1:**
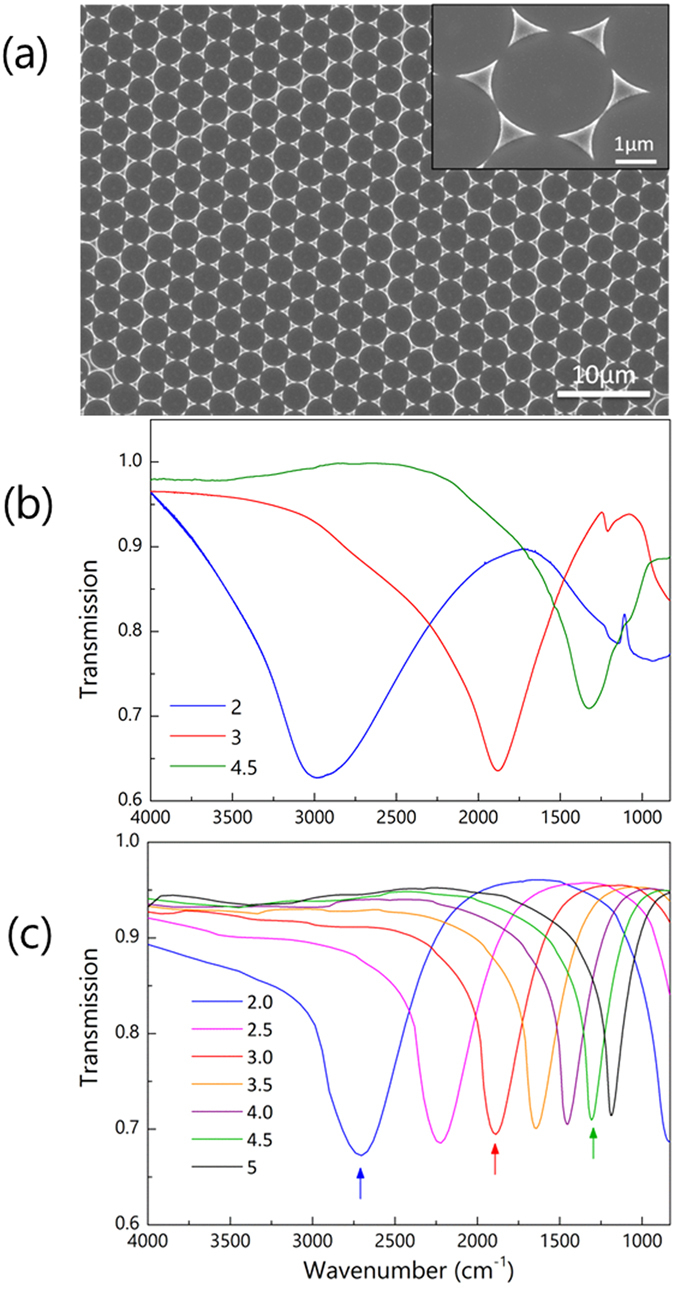
Tunable plasmon resonances of Al nanotriangles with different microspheres. (**a**) Scanning electron microscope (SEM) image of Al nanotriangles fabricated from 3 μm polystyrene spheres. The inset (30° tilted view) shows nanotriangles arranged in a hexagonal pattern. (**b**) Transmission spectra of Al nanotriangles fabricated from microsphere with different sizes (diameters). Bigger polystyrene microspheres results in bigger nanotriangles and thus resonances at longer wavelength range (smaller wavenumber). (**c**) Simulated plasmon resonances of Al nanotriangle arrays made from different sizes of microspheres. The three arrows indicate the three spectra corresponding to experimental results in panel (**b**).

In Fig. 1, we demonstrated this effective tuning mechanism with Al nanotriangles fabricated from 2, 3, 4.5 μm microspheres. Figure 1a shows the SEM image of an array of Al nanotriangles fabricated from 3 μm microspheres. In general, the nanotriangle arrays show good quality inherited from the removed microsphere monolayers. Defects can be observed in the SEM image as some nanotriangles are connected to each other instead of being well separated. Nevertheless, the nanotriangle arrays show well defined plasmon resonances as shown in Fig. 1b. Because of the large area of the nanoparticle arrays (~cm^2^), we can easily characterize the samples in a conventional FTIR spectrometer chamber. In our experiments, transmission of the nanotriangle arrays was measured and therefore the plasmon resonances are manifested as dips on the spectra. As shown in Fig. 1b, the plasmon resonance shifts from ~2970 cm^−1^ to ~1320 cm^−1^ as we change the sphere from 2 μm to 4.5 μm, covering a broad spectral range of molecular vibrations. For example, the characteristic absorption bands of -CH_2_ lie in the range between 2800 and 3000 cm^−1^ while proteins show fingerprint absorption in the range between 1500 and 1700 cm^−1^. Our experimental results show good agreement with the numerical simulations as shown in Fig. 1c, where other sizes of microspheres are included in the simulation demonstrating the facile tuning of the plasmon resonances of the nanotriangle arrays across a broad range simply by changing the size of the sphere. The discrepancy between the experiments and simulations, in terms of resonance frequency and linewidth, can be attributed to the size distribution of the Al nanotriangles and defects in the arrays, which arises from the imperfect microsphere monolayers.

The small spectral features around 1100 cm^−1^ superposed on the spectra are due to the excitation of surface phonon polaritons on the natural SiO_2_ layer as we will discuss later in the text. The polariton signal is the largest for Al nanotriangle arrays made from 2 μm microspheres while their major plasmon resonance is far away from this range. However, it is noted that additional plasmon resonance emerges in the longer wavelength (lower wavenumber) range providing better overlapping with the SiO_2_ surface phonon polariton frequencies. In addition, the number of sharp metallic tips or corners, where the enhanced near-fields are located, per unit area is larger for smaller microspheres, which also contributes to the larger polariton signals.

**Figure 2:**
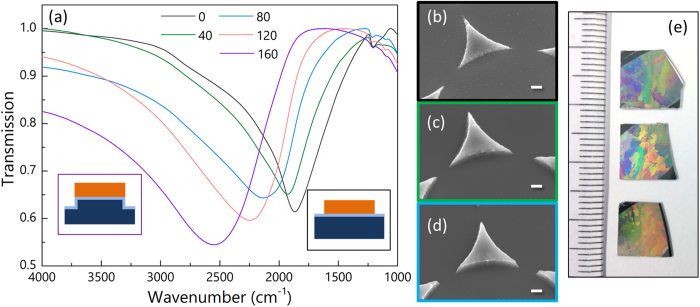
(**a**) Transmission spectra of the Al nanotriangles with different etching time (in seconds). The insets illustrate the etching effect on the substrate as well as the formation of SiO_2_ layer. (**b**–**d**) SEM images (30° tilted view) of three Al nanotriangles etched for 0, 80, and 160 seconds from top to bottom, respectively. (**e**) Optical images of the three samples shown in (**b**–**d**) (from top to bottom). The scale bars in (**b**–**d**) are 200 nm in length. The smallest scale on the ruler in (**e**) is 1 mm.

Using different microspheres provides a simple method to tune the plasmon resonances in a very broad range thanks to their ready availability. However, precise and continuous tuning of the resonance frequencies is desirable to achieve optimum spectral overlap with the molecular vibrations ensuring maximum plasmon enhancement. Etching of the substrates surrounding the nanoantennas presents an effective and controllable means to tune the resonance and the tuning magnitude can be accurately controlled by the etching time^34^,^35^. We employed this technique to further show the flexibility of colloidal lithography. We etched the Si substrates with CF_4_ plasma for different time and measured the transmission spectrum for each sample. Fig. 2 presents a summary for the etched Al nanotriangles made from 3 μm spheres.

Figure 2a shows the transmission spectra of the etched samples with different etching time. As expected, etching of the Si substrates leads to consistent blue-shift of the resonances due to the decreasing of the effective refractive index of the surroundings. Figure 2b–d show the morphology changes of the Al nanoparticles as they underwent CF_4_ plasma etching. It is clear that the etching shows good selectivity as the shape of the Al nanoparticles remains almost unchanged while the surrounding Si has been etched away. Closer examination of the SEM images reveals that the Al nanoparticle surfaces become smoother after the etching suggesting etching of Al occurred at the surface to some degree. Our measurements indicate an etching rate of 1.13 nm/s for Si, 1.26 nm/s for SiO_2_, and 0.06 nm/s for Al.

**Figure 3:**
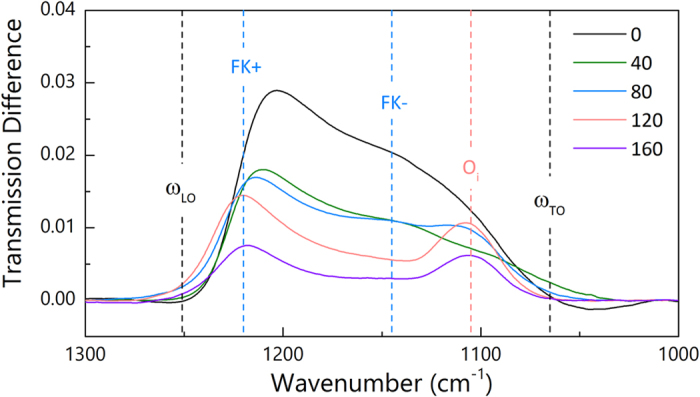
Baseline-corrected difference spectra of plasmon-enhanced surface phonon polaritons of the natural SiO_2_ layer. The vibration signals were extracted from the spectra in Fig. 2a. The color of the spectrum corresponds to that in Fig. 2a.

Besides the broad plasmon resonances from the Al nanotriangles, other absorption features are observed in the range of 1000~1300 cm^−1^, which are attributed to the excitation of surface phonon polaritons in the natural SiO_2_ layer as mentioned above. We fitted the spectra in that range with third order polynomials (Supplementary Information, Figure S1) and calculated difference between the original spectra and the fitted baseline. Figure 3 shows the difference signals of the SiO_2_ vibrations after baseline corrections. The vibration frequencies lie in between the typical transverse optical (TO) phonon frequency ω_TO_ and longitudinal optical (LO) phonon frequency ω_LO_ of SiO_2_ indicating the excitation of surface phonon polaritons. Two types of phonon polaritons were observed in this range as indicated in Fig. 3 as FK+ and FK−, which correspond to Fuchs-Kliewer (FK) modes at the surface (SiO_2_/air) and the interface (Si/SiO_2_), respectively^35^,^36^. Without any etching, both the FK+ and FK- modes show the highest signals. As etching increase, the peak intensity drops. For the cases of 120 and 160 seconds of etching, only FK+ mode is observed. Here, two factors affect the shape and the intensity of the two polariton signals: the plasmon resonances and the removal of the Si substrates. As shown in Fig. 2, etching of the Si substrate progressively shifts the plasmon resonances to higher wavenumber range moving away from the FK phonon polariton range. Therefore, the plasmon enhancement gradually becomes weaker. Furthermore, SiO_2_ had also been removed together with Si during the etching. Although new layer of SiO_2_ grew again once the samples were exposed to air, the newly-formed SiO_2_ located further away from the plasmonic hot spots. Therefore, the spectral change shown in Fig. 3 is a combined effect of these two factors. The initial etching from 0 to 40 seconds resulted in the biggest intensity drop of the two modes.

It is noted that peak at 1105 cm^−1^ is attributed to the vibration signals from the interstitial oxygen atoms (O_i_) in the Czochralski (CZ) method prepared Si wafers^37^.

**Figure 4:**
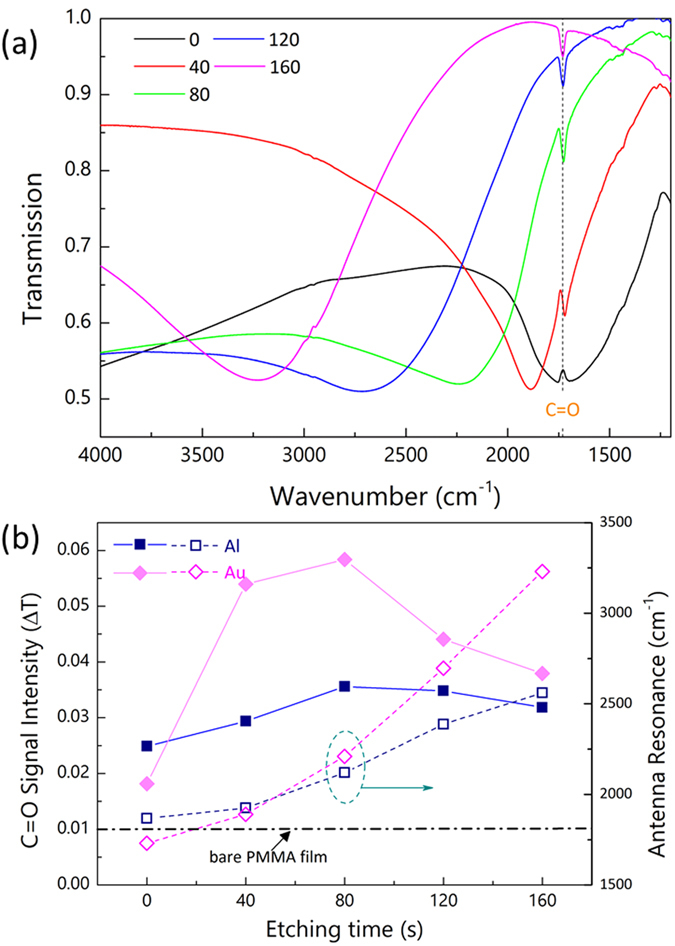
(**a**) Transmission spectra of Au nanotriangle arrays with different etching time after thin PMMA coating. (**b**) Comparison of the C=O vibration signals (solid symbols, left axis) from Al and Au nanotriangle arrays with different etching time. The signal from the bare PMMA thin film (dash dot) is also shown for comparison. The antenna resonance frequencies (hollow symbols, right axis) of Al and Au nanotriangles are also shown as a function of etching time.

So far, we have introduced the scalable fabrication of large area Al nanotriangles using microspheres and demonstrated two different tuning methods to accurately control the nanoantenna resonances: using of different microspheres and post-fabrication etching. We utilized such facile tuning methods and investigated their performances as SEIRA substrates. Recently, Al nanostructures or metamaterials have received a lot of attention to study the possibility of using Al as alternative plasmonic materials^10^,^38^,^39^,^40^. Here we directly compared the SEIRA signals from Poly(methyl methacrylate) (PMMA) thin films coated on top of Al and Au nanotriangles and the results are shown in Fig. 4.

We fabricated Au nanotriangles using 3 μm microspheres same as the case of Al nanotriangles. Thin PMMA films were coated onto those substrates via spin-coating. We use the vibration signals of the carbonyl band C=O in the PMMA as an indicator of the SEIRA performance. Figure 4a shows the raw transmission spectra of the Au nanotriangles after PMMA coating. With no etching, the Au nanoantenna resonance perfectly overlaps with the C=O vibration which appears on the spectrum as anti-resonance^1^. With 40 s etching, the C=O vibration signal assumes a strong Fano shape due to the strong coupling between the plasmon mode and the C=O vibration. Further etching moves the antenna resonance away from the C=O vibration frequency leading to weaker coupling and the signal resumes a Lorentz-like shape.

Similar to Fig. 3, we fitted the raw spectra in the range of 2000–1400 cm^−1^ with third order polynomials and calculated difference between the original spectra and the fitted baseline (Supplementary Information, Figure S2a). The antenna-enhanced C=O vibration signals from both Al and Au nanotriangle arrays were directly compared in Fig. 4b. Here, the “C=O Signal Intensity” refers to the peak-to-dip amplitude of the vibrational signal (Supplementary Information, Figure S2b). The SEIRA signals depend on the two factors introduced by the etching. The etching enables continuous tuning of the antenna resonances in one direction toward higher wavenumber (shorter wavelength) as shown in Fig. 4b. This blue-shift of the resonance modulates the coupling strength and thus the plasmon-enhanced vibration signals. Unlike in Fig. 3, where the etching removes SiO_2_ materials reducing the phonon-polariton intensity, the partial removal of the substrates here provides larger accessible enhanced near-field volume to the PMMA molecules. Therefore, the etching here offers big advantages for molecular sensing as demonstrated in Fig. 4b.

For both Al and Au nanotriangle arrays, the C=O signal intensity increases first with etching time and then drops after 80 seconds. The signal modulation can be attributed to the combined effect of increased sensing volume together with the detuned plasmon resonances. Although the etching continuously shifts the plasmon modes away from the C=O vibration frequency, it also frees enhanced near-field from the Si substrates and enables more molecules packed in the sensing volumes. In particular, with 40-second etching, the signal from Au nanotriangle arrays increases nearly 3 times. At 80 seconds, the etching depth is estimated to be ~90 nm, which is close to the spatial extent (~100 nm) of vibrational signal enhancement^35^. Therefore, further etching does not help to increase the effective sensing volume but only leads to additional blue-shift of the antenna resonance. Therefore, the signal strength gradually decreases after 80 seconds due to the increasingly poor spectral overlap between the antenna resonances and the vibration frequency.

**Figure 5:**
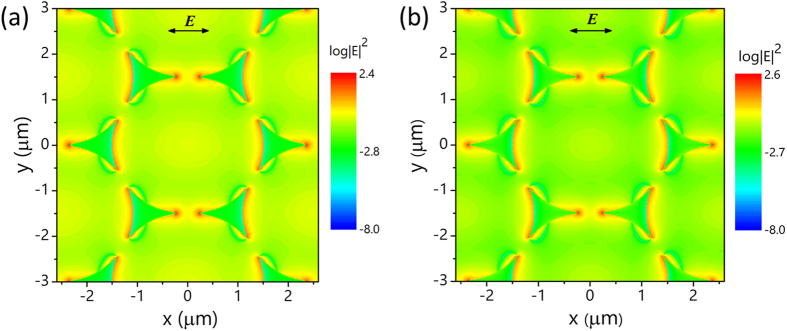
Electric near-field profile of the etched (**a**) Al nanotriangles and (**b**) Au nanotriangles. The substrate is assumed to be etched 40 nm in depth. The two types of nanoantennas show similar near-field enhancement with slightly higher intensity for Au nanoantennas.

The effect of etching is more pronounced on the Au nanotriangle arrays than on the Al nanotriangle arrays. We attribute this to the existence of natural Al_2_O_3_ oxide layers (2–3 nm in thickness) encapsulating the Al nanotriangles. Both the etched Al and Au nanotriangles show similar enhanced near-field intensities as shown in Fig. 5. However, for Al nanotriangles, the existence of Al_2_O_3_ oxide layer keeps the molecules from the highest enhanced near-field near the metal surface. Therefore, for molecular sensing applications, Au offers better sensitivity and stability while Al is more preferable in terms of material cost. In addition, the Al_2_O_3_ layer weakened the drastic changes of the surroundings by etching and thus resonance shift of Al nanotriangles is not as significant as that of Au nanotriangles as shown in Fig. 4b.

In conclusion, we have used colloidal lithography with microspheres to fabricate Al and Au nanotriangle arrays. In addition to the wide resonance tuning by using different sizes of microspheres, we demonstrated effective and continuous tuning by post-fabrication dry etching of the Si substrates with the metal nanotriangles as masks. This post-processing step not only provides an effective means to tune the resonance but also increases the accessible enhanced near-field volume. We directly compared the SEIRA performance of Al and Au nanotriangle arrays with different etching time confirming that better molecular sensitivity can be achieved with the etching process. Regarding the signal strength for molecular sensing, gold is still the material with larger enhancement and better chemical stability while aluminum provides promising alternative choices for applications where cost is the priority.

## Methods

### Sample Preparation

The formation of the deposition masks of colloidal sphere monolayers followed our previous work. In brief, polystyrene microspheres (2, 3, and 4.5 μm in diameter, Polysciences Inc.) were dispersed in a water/ethanol mixture (1:1 volume ratio). The microspheres were then spread out on the water/air interface forming a close-packed monolayer. The monolayer was later transferred onto a Czochralski (CZ) Si substrate. Al and Au were deposited onto the substrates by electron beam deposition followed by removal of the microspheres in toluene with ultrasonication. For Au deposition, a thin Ti film of 5 nm was used as the adhesion layer. For SEIRA studies, diluted PMMA solutions were prepared with PMMA and thinner in a volume ratio of 1:4 and then thin PMMA films were deposited onto the samples via spin-coating at 6000 rpm.

### Dry Etching

The etching of Si substrates was achieved with CF_4_ gas in an ICP-RIE system (ICP: interactively coupled plasma; RIE: reactive-ion etching; CE-300I, ULVAC Inc.). The flow rate of the CF_4_ gas is 20 sccm. The antenna RF power is 200 W and the bias RF power is 20 W. The etching effect was examined using a Hitachi FE-SEM (SU8200).

### FTIR Measurement

The transmission spectra of the samples were recorded with an FTIR spectrometer (Nicolet iS50, Thermo Fisher Scientific Inc.). During the measurement, the spectrometer chamber was constantly purged with high-purity N_2_ gas. The transmitted light through the samples was collected using a liquid-nitrogen-cooled mercury cadmium telluride (MCT) detector. The transmittance was calculated using a plain Si chip as the reference.

### Numerical Simulation

The transmittance spectra and the electric field distributions of the plasmonic triangles arrays were calculated using the rigorous coupled-wave analysis (RCWA) (DiffractMOD, RSoft) and the finite-difference time-domain (FDTD) (FullWAVE, RSoft) methods, respectively. The dielectric functions of Au, Al, Al_2_O_3_, SiO_2_ and Si were taken from the literature^41^. For both RCWA and FDTD simulations, the excited electromagnetic field propagated along the -*z* axis and the electric field oscillated along the *x* axis. The incident electric field amplitude was normalized to 1.

## Additional Information

**How to cite this article:** Chen, K. *et al*. Tunable Nanoantennas for Surface Enhanced Infrared Absorption Spectroscopy by Colloidal Lithography and Post-Fabrication Etching. *Sci. Rep.* **7**, 44069; doi: 10.1038/srep44069 (2017).

**Publisher's note:** Springer Nature remains neutral with regard to jurisdictional claims in published maps and institutional affiliations.

## Supplementary Material

Supplementary Information
