# Supplementary material for: Tunable Nanoantennas for Surface Enhanced Infrared Absorption Spectroscopy by Colloidal Lithography and Post-Fabrication Etching
Source: Sci Rep. 2017 Mar 8;7:44069. doi: 10.1038/srep44069 (PMC5341049; doi:10.1038/srep44069)
Supplement: Supplementary Information [file srep44069-s1.doc]

Tunable Nanoantennas for Surface Enhanced Infrared Absorption Spectroscopy by Colloidal Lithography and Post-Fabrication Etching

Kai Chen^1,2*^, Thang Duy Dao^1,2^, and Tadaaki Nagao^1,2,3*^

^1^ International Center for Materials Nanoarchitectonics (MANA), National Institute for Materials Science (NIMS), 1-1 Namiki, Tsukuba, Ibaraki 305-0044, Japan

^2^ CREST, Japan Science and Technology Agency, 4-1-8 Honcho, Kawaguchi, Saitama, 332-0012, Japan

^3^ Department of Condensed Matter Physics, Graduate School of Science, Hokkaido University, Sapporo 060-0810, Japan

*[chen.kai@nims.go.jp](mailto:chen.kai@nims.go.jp); [nagao.tadaaki@nims.go.jp](mailto:nagao.tadaaki@nims.go.jp)


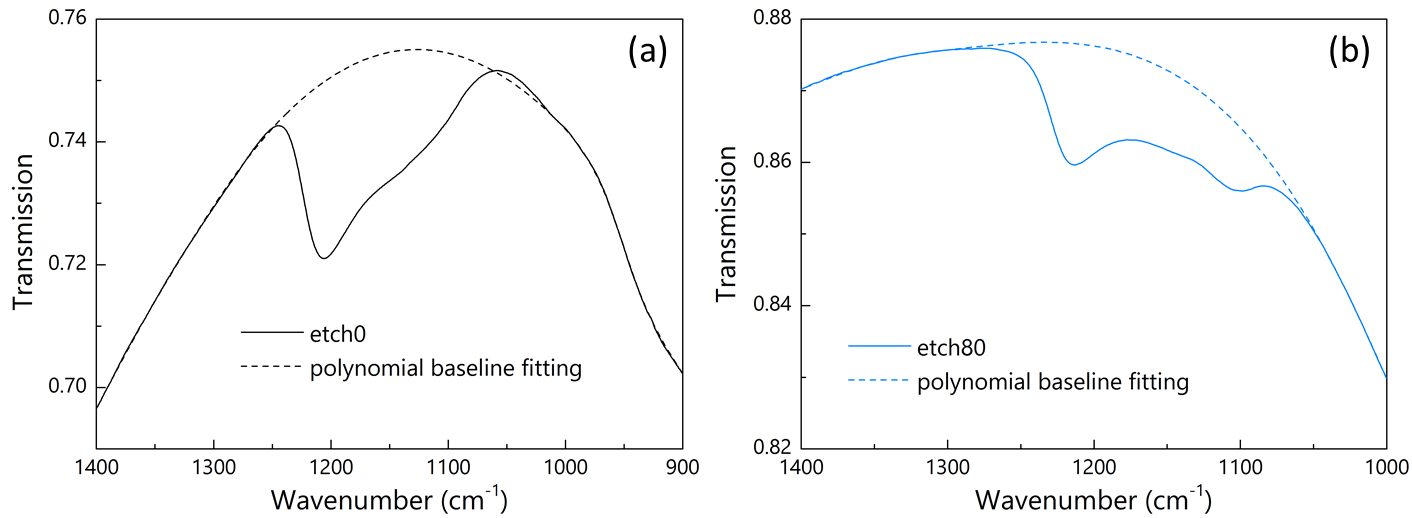


**Figure S1**. The transmission spectra (solid) and the corresponding polynomial baseline (dashed) for the Al nanotriangle arrays (a) without any etching and (b) with 80 second of etching. The two samples are selected from Fig. 2 in the main text. The line color thus corresponds to that used in Fig. 2. The spectral range was selected to cover the frequencies of surface phonon polaritons of the SiO_2_ layer.


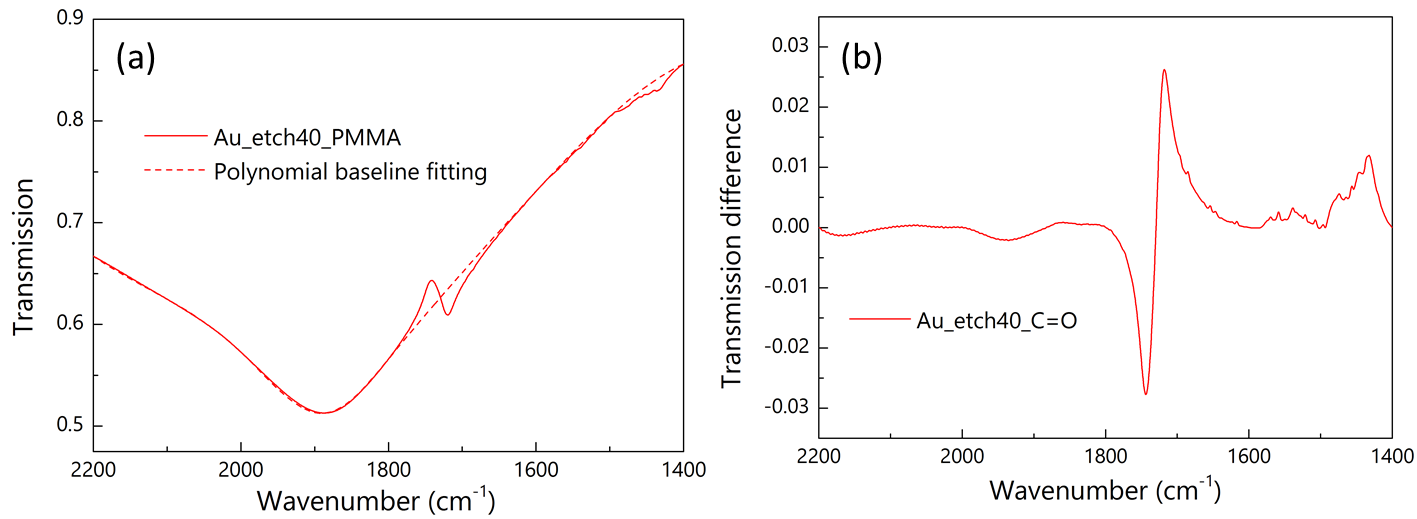


Figure S2. (a) The transmission spectrum of Au nanotriangle arrays with 40 second of etching after PMMA thin film coating (solid line) and the 3^rd^ order polynomial fitted baseline (dashed). (b) The difference spectrum is calculated from panel *a* and the peak-to-dip amplitude is used as the signal intensity.
